# Bibliographical

**Published:** 1883-04

**Authors:** 


### Bibliographical.

*The Science and Practice of Medicine; or, the Pathology and Therapeutics of Internal Diseases.* Alonzo B. Palmer, M. D., LL.D., Professor of Pathology and Practice of Medicine and of Clinical Medicine in the University of Michigan. New York: G. P. Putnam's Sons, 1882, 2 volumes.

Prof. Palmer's two volumes on the Practice of Medicine have already, vulgarly speaking, "been through the mill," and one or two of the mills had decidedly hard and *very* sharp teeth. An American undertaking it is; but not the first. Other practitioners, some in New York City, some elsewhere, have written books on Practice, in part, at any rate, based upon their own experiences in hospitals and asylums. We agree with the views of a correspondent, whose views are given in a communication elsewhere in The Independent Practitioner. But then he does drive a little too hard against publishers, who, in reality, have done something besides publishing reprints and translations of foreign authors.

What to say about Prof. Palmer's book? First, what is his aim? It seems to us to be to tell Americans just how to treat diseases that he meets with in every-day practice, without dwelling very particularly upon the pathology lesions which, whether recognized or not, lie between the lines of nearly every prescription that evidences a plan of treatment.

Therapeutics can never take precedence of pathology, any more than a varnish can restore the rottenness underneath. A man learns a great deal of therapeutics after he leaves Alma Mater. He does not so often imbibe pathological knowledge. Thus "specifics" are not so much thought of the second and third year after graduation as they were at the lecture, and the opinion of them declines steadily thereafter.

Pathology and pathogenesis are first to be clearly marked in the memory; then are the symptoms full of meaning and the use of drugs full of logic. A prescription "learned and conned by rote" is about on a par with reading prayers from the pulpit from a popular printed collection of them.

What Prof. Palmer has done is to give a fairish statement of symptoms, a meagre pathology and a very comprehensive line of treatment.

His Latin and his English have been criticised by the medical press. No doubt a second edition will correct all the errors in grammar and construction. It does not serve our purpose to put in any of his odd phraseology or faulty prescriptions for the amusement of our readers. Oftentimes a disease, a set of diseases, is not well done. Notably, the spinal cord and the liver are not written up to the times. Polymyelitis we find ; *Polio* is the term. We can only hope that the true meaning and etymology were known, and that the unconscious printer was to be blamed.

The classification could be better, and it seems to us that the matter could be more compact and condensed, both in expression and on the printed page. The treatment, however, gives many a hint that will be accepted and acted upon by those whose experience is less than Dr. Palmer's. The style of paper and of type is excellent, the usual work of the Putnams.

*Bromide of Ethyl*, the most perfect anæsthetic for short, painful surgical operations. By JULIAN J. CHISHOLM, M. D., Professor of Eye and Ear Diseases in the University of Maryland.

This is a reprint from the *Maryland Medical Journal*, and details the experience of the author with the agent in question. His estimate of the virtues may be gathered from the title of the paper. He says: "Its wonderful action is obtained during the first minute of its inhalation, and what I have called its primary anæsthesia." Accordingly he pushes the administration to the utmost, excluding the air as far as possible, and claims to produce perfect anæsthesia in from twenty to sixty seconds, without nausea or succeeding dullness. His directions are as follows: "The best inhaler for the giving of bromide of ethyl is a thick towel folded into the form of a small cone, with a closed apex. Between one of the folds of this towel I place a sheet of paper, which makes the cone nearly air-tight. The base of the cone must be wide enough to inclose both mouth and nose. I instruct the patient how to make long inspiration, and inform him he must do this notwithstanding the fact that he will feel somewhat stifled. The cone must not be removed from the face for an instant until anæsthesia is produced. I have found this anæsthetic sleep to last not more than two or three minutes, often not so long."

This method of administration is very well in giving nitrous oxide, which is very weak in anæsthetic power, but bromide of ethyl appears to be more potent. Certainly, in our experiments upon some of the lower animals we have produced a narcosis as profound as that of ether, and seemingly as lasting. But the symptoms attending its use were frequently so alarming that we have never dared give it to a human being. Clonic, and even tetanic convulsions, extreme nausea, and subsequent depression, were indications of too profound disturbances for perseverance in its use. It was not, however, administered according to the directions of Dr. Chisholm, but was given slowly and carefully. The method of placing a stop-watch in the hands of an assistant to determine in just how many seconds anæsthesia may be reached and an operation performed does not commend itself to a careful man, who is usually quite content to occupy a little more time in the hope and expectation that additional security may be obtained.

*Headaches:* Their Nature, Causes and Treatment. Wm. H. Day, M. D. Philadelphia: P. Blakiston & Co. 1883.

The subject of "Headache" interests physician and patient alike. Oftentimes one would rather "strike" a case of measles than treat a case of headache that the patient—usually female—"has had on and off for years."

The whole subject is concisely and completely covered in this work, and is equally readable by the laity and the profession. It is a book to be recommended in all respects *per se*; but we wish the paper were a little better than ordinary newspaper.

*Alcoholic Inebriety:* From a Medical Standpoint. Joseph Parish, M.D. Philadelphia: P. Blakiston, Son & Co. 1883. $1.25. An interesting and thoughtful monograph.

